# Accelerometer-measured absolute versus relative physical activity intensity: cross-sectional associations with cardiometabolic health in midlife

**DOI:** 10.1186/s12889-023-17281-4

**Published:** 2023-11-24

**Authors:** Jonatan Fridolfsson, Daniel Arvidsson, Elin Ekblom-Bak, Örjan Ekblom, Göran Bergström, Mats Börjesson

**Affiliations:** 1 Center for Health and Performance, Department of Food and Nutrition and Sport Science, Faculty of Education, University of Gothenburg, Box 300, 405 30 Gothenburg, Sweden; 2 Department of Physical Activity and Health, Swedish School of Sport and Health Sciences, Stockholm, Sweden; 3 Department of Molecular and Clinical Medicine, Institute of Medicine, Sahlgrenska Academy, University of Gothenburg, Gothenburg, Sweden; 4 Department of Clinical Physiology, Region Västra Götaland, Gothenburg, Sweden; 5 Center for Lifestyle Intervention, Department of Molecular and Clinical Medicine, Institute of Medicine, Sahlgrenska Academy, University of Gothenburg, Gothenburg, Sweden; 6 Sahlgrenska University Hospital, Region Västra Götaland, Gothenburg, Sweden

**Keywords:** Cardiometabolic risk factors, Cardiovascular disease, Cardiovascular fitness, Recommendations

## Abstract

**Background:**

Observational studies investigating the association between accelerometer-measured physical activity and health all use absolute measures of physical activity intensity. However, intervention studies suggest that the physical activity intensity required to improve health is relative to individual fitness. The aim of this study was to investigate the associations between accelerometer-measured absolute and relative physical activity intensity and cardiometabolic health, and what implications these associations may have on the interpretation of health-associated physical activity.

**Methods:**

A sample of the cross-sectional Swedish CArdioPulmonary bioImage Study (SCAPIS) consisting of 4,234 men and women aged 55–64 years was studied. Physical activity intensity was measured by accelerometry and expressed as absolute (e.g., metabolic equivalents of task) or relative (percentage of maximal oxygen consumption). Fitness was estimated by the submaximal Ekblom-Bak test. A composite (‘metabolic syndrome’) score combined measures of waist circumference, systolic blood pressure, high-density lipoprotein, triglycerides, and glycated hemoglobin. Associations of absolute and relative physical activity intensity with the health indicators (i.e., fitness and metabolic syndrome score) were studied by partial least squares regression. Analyses were stratified by fitness level.

**Results:**

Both absolute and relative physical activity intensity associated with the health indicators. However, the strongest associations for absolute intensity varied depending on fitness levels, whereas the associations for relative intensity were more synchronized across fitness groups. The dose–response relationship between moderate-to-vigorous intensity and the health indicators was stronger for relative than for absolute intensity. The absolute and relative moderate-to-vigorous intensity cut-offs intersected at the 5th fitness percentile, indicating that the absolute intensity cut-off is too low for 95% of individuals in this sample. While 99% of individuals fulfilled the general physical activity recommendations based on absolute intensity measures, only 21% fulfilled the recommendations based on relative intensity measures. In relation to a “sufficient” fitness level, 9% fulfilled the recommendations.

**Conclusions:**

Accelerometer-measured relative physical activity intensity represents the intensity related to health benefits regardless of fitness level. Traditional absolute moderate intensity accelerometer cut-offs are too low for most individuals and should be adapted to the fitness level in the sample studied. Absolute and relative physical activity intensity cannot be used interchangeably.

**Supplementary Information:**

The online version contains supplementary material available at 10.1186/s12889-023-17281-4.

## Background

The health benefits of physical activity (PA) are related to the intensity and volume of activity [1]. PA intensity can be expressed in absolute or relative terms [2]. Absolute PA intensity measures include energy expenditure, locomotion speed and mechanic work, all of which represent the same absolute intensity regardless of who is performing the activity. Relative PA intensity refers to absolute PA intensity in relation to individual maximal PA capacity and differs depending on individual cardiorespiratory fitness. Cardiorespiratory fitness refers to the maximal oxygen consumption of an individual. Examples of relative intensity measures are proportion of maximal oxygen consumption, proportion of maximal heart rate, and self-perceived exertion.

PA intensity is today typically measured by accelerometers. The acceleration measured is closely related to mechanical workload and is an absolute measure of PA intensity [3]. The absolute acceleration output is often translated to absolute energy expenditure, based on calibration studies using indirect calorimetry as reference [4]. This continuous estimate of energy expenditure is then used to determine the time spent at different absolute intensity levels. Although PA intensity is measured in absolute terms, it is often expressed in relative terms (as light, moderate, and vigorous). In absolute terms, sedentary is defined as time spent at an absolute energy expenditure below 1.5 metabolic equivalents of task (METs), where 1 MET represents an oxygen consumption of 3.5 mL/min/kg. Absolute light, moderate, vigorous and very-vigorous intensity is defined as time spent above 1.5, 3, 6 and 9 METs respectively [2]. The resulting time spent at these intensity levels is used in observational studies to investigate associations with health and fulfilment of recommendations on PA [1].

Relative PA intensity can be determined by the oxygen consumption of an activity in relation to individual maximal oxygen consumption and is defined as moderate intensity above 46%, vigorous above 64% and very-vigorous above 91% [5]. For the moderate intensity cut-off, the absolute 3 METs is equivalent to the relative 46% only in individuals with a maximal oxygen consumption of 22.8 mL/min/kg [5]. From a relative perspective, 3 METs would be too low for individuals with higher fitness level than 22.8 mL/min/kg, and too high for individuals with lower fitness. This mixture of absolute and relative PA intensity in the use of accelerometer data to determine PA levels causes confusion and misunderstanding in the interpretation of the results, and in the evaluation of the importance of PA to health.

The current consensus in PA research is that the main health benefits of PA come from time spent at moderate-to-vigorous intensity [2]. Recommendations state that adults should undertake 150–300 min per week of moderate intensity PA, or 75–150 min per week of vigorous intensity, or an equivalent combination of both [1]. The PA guidelines suggest that absolute or relative intensity can be used interchangeably for most individuals with the exception of older adults with low fitness level, for whom relative intensity is more appropriate [1, 2]. However, fitness levels vary considerably between individuals and also decrease with age [6]. This suggest absolute and relative intensity should not be used interchangeably. In addition, PA recommendations are mainly based on self-reported PA, which lack detail about intensity [4].

To understand how the different intensity measures should be used, their association with health outcomes must be studied in more detail by objective measures. The association between PA and cardiometabolic health is of particular interest due to the importance of PA to reduce the risk of cardiovascular disease. [7] The association between PA and cardiometabolic health is considered to be both moderated and mediated by fitness [2]. The moderation by fitness refers to the association between PA and cardiometabolic health being different depending on fitness level. The mediation by fitness refers to the association between higher PA and better cardiometabolic health being caused by an increase in fitness level.

Furthermore, there is conflicting evidence from previous studies as to whether absolute light, moderate or vigorous intensity is required for health benefits. In older individuals, associations with cardiometabolic health can be found at absolute light intensity, whereas in younger individuals absolute vigorous intensity may be required for any significant associations with cardiometabolic health [8–10]. The age differences between these samples could be considered a proxy for differences in fitness level. Relative intensity was not considered in these highly diverse study samples, which might be a contributing factor to the lack of consensus.

Previous studies have demonstrated that accelerometer-measured absolute PA intensity can be translated to relative intensity [11–15]. This is done by applying available calibration equations to estimate oxygen consumption from accelerometry data, and then relating the estimated oxygen consumption to the measured fitness level. However, the health benefits of accelerometer-measured relative PA intensity have not been investigated. The purpose of this study was therefore to investigate the associations of accelerometer-measured absolute and relative PA intensity with cardiometabolic health, and what implications these associations may have on the interpretation of health-associated PA.

## Methods

### Study sample

A sample of the Swedish multicenter observational study SCAPIS (Swedish CArdioPulmonary bioImage Study) [16] was analyzed. SCAPIS includes 30 154 randomly selected men and women aged 50–64 years, with objectively measured PA and extensive measurements of markers of cardiovascular health [16, 17]. Cardiorespiratory fitness was estimated in a subsample from the study center in Gothenburg. All participants with estimated fitness, valid PA measurements, and measurements of cardiometabolic health indicators were included in this study (*n* = 4 176). The data collection was carried out in 2013–2018. SCAPIS has been approved by the ethics committee at Umeå University (no. 2021–228-31 M) and the current study has received specific approval by the Regional ethical board in Gothenburg (no. 638–16). Written, informed consent was retrieved from all participants.

### Physical activity and fitness

Participants wore a triaxial accelerometer (ActiGraph model GT3X + , wGT3X + or wGT3X-BT, ActiGraph, Pensacola, Florida, USA) in an elastic belt over their right hip for seven continuous days. They were instructed to take the accelerometer off during sleep and water-based activities. Raw accelerometer data were extracted and processed using the 10 Hz frequency extended method (FEM) [18]. This method enables more detailed and accurate measurement of PA intensity compared to the most commonly used processing method using ActiGraph counts [3, 18, 19]. Triaxial accelerometer data were combined to a vector magnitude and reduced to 3 s epochs. Non-wear time was defined as 60 min of zero accelerometer output with allowance of up to 2 min of interruptions below the sedentary threshold [20]. A valid day was defined as at least 10 h of wear time and a valid measurement as at least 4 valid days [21]. The variables retrieved from the processed accelerometer data represent time spent at different intensity levels.

In this study, absolute PA intensity was expressed as metabolic equivalents of task (METs), oxygen consumption and locomotion speed [18, 22]. The definition of one MET is the oxygen consumption during rest, generally considered to be 3.5 mL/min/kg [2]. On an absolute scale, PA intensity is generally defined as sedentary (< 1.5 METs), light (≥ 1.5- < 3 METs), moderate (≥ 3- < 6 METs), vigorous (≥ 6- < 9 METs) and very-vigorous (≥ 9 METs) [2, 21], which correspond to an oxygen consumption of < 5.25, ≥ 5.25- < 10.5, ≥ 10.5- < 21.0, ≥ 21.0- < 31.5 and ≥ 31.5 mL/min/kg respectively. This study expressed relative PA intensity as proportion of estimated maximal oxygen consumption during activity, standardized to bodyweight. On a relative scale, PA intensity is defined as light (< 46%), moderate (≥ 46- < 64%), vigorous (≥ 64- < 91%) and very-vigorous (≥ 91%) of maximal oxygen consumption according to the American College of Sports Medicine [5].

Maximal oxygen consumption (referred to as fitness) was estimated by the Ekblom-Bak submaximal cycle ergometer test [23]. The testing procedure include cycling at two submaximal workloads, the first at light intensity and the second at estimated moderate intensity, while the heart rate response is measured. The ratio of the difference in heart rate and the difference in workload is calculated and compared to published reference values. The Ekblom-Bak test has high validity as reference to direct measurement with cross validated R^2^adj = 0.90 and standard error of estimate: 0.30 L/min for all ages, and R^2^adj = 0.84 and standard error of estimate: 0.33 L/min for 50–64 years old [23]. Exclusion criteria for the fitness test were ongoing infections, known unstable cardiovascular disease, indication of cardiac disease from electrocardiography patterns, medication with beta-blockers, weight above 125 kg or resting heart rate above 100 beats per minute. In addition, participants who refrained from performing the fitness test was not tested.

Processed accelerometer output was used to estimate the time spent at different absolute and relative PA intensity levels [3, 18]. A detailed PA intensity spectrum consisting of 22 intensity variables was generated to represent an absolute PA intensity pattern [19, 24]. The bin edges dividing the PA intensity spectrum variables were 0, 40, 80, 160, 240 mg, and then increasing in 80 mg intervals to 1600 mg and above. The same intensity spectrum was used to represent relative PA intensity by first translating the spectrum cut-offs from accelerometer output to oxygen consumption, and then dividing by estimated maximal oxygen consumption. Translation was done by a linear regression model based on published reference values [18]. The regression coefficients were Y = 0.02683X + 5.108, where Y represents oxygen consumption in mL/min/kg and X represents processed accelerometer output in mg. Traditional crude cut-points representing sedentary, light-, moderate-, vigorous- and very-vigorous PA were used for reference [5, 18].

### Metabolic syndrome score

To provide an overall measure of individual cardiometabolic health, a metabolic syndrome score was used [25, 26]. The metabolic syndrome refers to the clustering of several cardiometabolic risk factors, including central obesity, dyslipidemia, hyperglycemia and elevated blood pressure, and can be used to predict the risk of future cardiovascular disease [27]. Central obesity was represented by waist circumference, dyslipidemia by high-density lipoprotein (HDL) and triglycerides, hyperglycemia by glycated hemoglobin (HbA1c), and hypertension by systolic blood pressure (SBP) [27]. Waist circumference was measured by measuring tape according to standardized methods [28]. A venous blood sample was collected after an overnight fast and was used for measuring HDL, triglycerides and HbA1c levels. SBP was measured twice in each arm by an automated device (Omron M10-IT, Omron Health care Co, Kyoto, Japan) and the mean of the measurements was used. Examinations were performed on two or three occasions within two weeks. All variables were measured within two weeks (at the examinations before or after the week of accelerometer use).

All measured risk factors have a positive association with cardiovascular disease (e.g. higher values are associated with higher risk of disease), except HDL which has a negative association with cardiovascular disease. Therefore, HDL was multiplied by -1 to have a positive association with the other risk factors. For dyslipidemia to not be more influential in the composite score due to more variables, the mean of sex standardized Z-scores of HDL and triglycerides was first calculated [26]. Subsequently, the mean of sex standardized Z-scores of waist circumference, HbA1c, SBP and the combined value of HDL and triglycerides was calculated as the metabolic syndrome composite score.

### Statistical analyses

To investigate differences in PA intensity depending on fitness, analyses were stratified by fitness level. Fitness was standardized by sex and used for dividing the sample into tertiles. Differences between fitness groups in risk factors and absolute and relative crude PA levels were investigated by ANOVA with post hoc t-tests. Detailed absolute and relative PA intensity patterns were visualized by standardizing the PA volume in the intensity spectrum variables in the whole study sample to a mean of zero and a standard deviation of one. Standardization was performed by subtracting the mean and dividing by the standard deviation for each intensity variable. Subsequently, the mean of each standardized variable was presented for each fitness group [24].

The proportion of individuals fulfilling the PA recommendations of 150 min of moderate-to-vigorous PA [1] was calculated using both absolute and relative PA measures. Previous research has suggested a maximal oxygen consumption capacity of 31.5 and 35 mL/min/kg for women and men, respectively, to be a sufficiently high fitness level to achieve most potential health benefits [29]. The proportion of individuals fulfilling the PA recommendations with a moderate-to-vigorous intensity cut-off in relation to a sufficiently high fitness level was calculated. The 46% moderate-to-vigorous intensity cut-off for an individual with this fitness level is equivalent to an oxygen consumption of 14.5 and 16.1 mL/min/kg for men and women, or 4.1 and 4.6 METs, respectively.

Since the variables representing the PA intensity spectrum are highly colinear, partial least squares regression (PLS) was used to investigate the association with fitness and metabolic syndrome score [30, 31]. Separate PLS models were used to investigate the associations of absolute and relative PA intensity in the whole study sample and in the 3 fitness groups. If the variables were skewed, they were square root transformed. The number of latent variables (PLS components) was selected based on Monte Carlo resampling with 10^3^ repetitions and a cut-off of a quarter of a standard deviation, to ensure that the PLS model was significantly better than a model with fewer components [32].

Selectivity ratio plots were used to represent the contribution of each PA intensity spectrum variable to the association with the outcome [33]. The selectivity ratio represents the explained variance in the PA spectrum variables from the latent variables. To get an estimate of the explained variance in the health variable, the selectivity ratio is multiplied by the overall explained variance of the PLS model. However, the explained variance in the outcome by a single intensity level should not be interpreted independently, since the results from the PLS regression represent a pattern of the PA variables combined rather than independent associations with the separate variables.

The 95% confidence interval of the selectivity ratio was calculated by bootstrapping and the statistical uncertainty of the PLS model was assessed by permutation tests, both with 10^4^ repetitions [31]. Furthermore, the PA intensity level where less than one third of the individuals had any movement was indicated as dashed lines in the figures. In addition, to facilitate interpretation of the results from the more advanced analyses, the dose–response association between absolute and relative moderate-to-vigorous PA and the metabolic syndrome score and fitness was calculated using linear regression. All data processing and statistical analyses were performed in MATLAB 2022a (MathWorks, Natick, MA, USA).

## Results

### Characteristics and group differences

The number of participants at the Gothenburg study site was 6 266, and the number of participants with valid fitness tests were 4 513. Of the participants with valid fitness tests, 4234 had valid accelerometer measured PA. The number of participants with measurements of fitness, PA, and metabolic syndrome score, and thus used in all further analyses, was 4 176. The 4 176 participants with valid data had on average more favorable cardiometabolic health indicators compared to the excluded participants from the Gothenburg study site as well as compared to the entire SCAPIS sample. The group differences are presented in the Supplementary Table 1 in the Additional file 1.

**Fig. 1:**
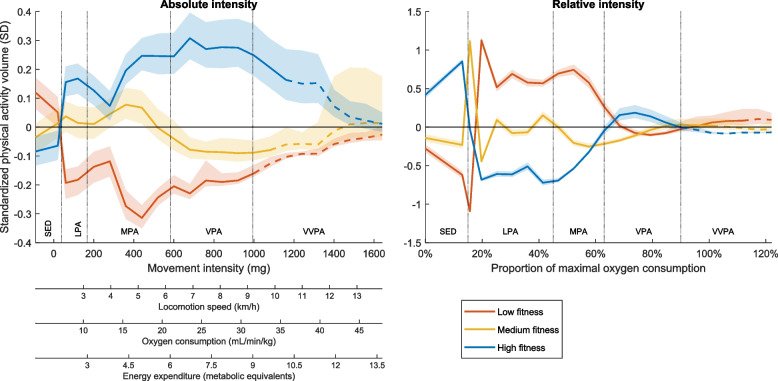
Fitness-group means of standardized PA intensity spectrum represented as absolute (left) and relative mean group fitness (right). Values are calculated in relation to total sample mean and standard deviation where 0 represents the mean and 1 the standard deviation for each of the PA intensity spectrum variables. Dashed lines indicate where less than one third of the individuals had any movement. Shaded areas represent 95% confidence intervals. Since no established relative cut off for sedentary is available, the absolute cut off at 1.5 METs was used as reference for relative sedentary time based on the average fitness in the sample. SD standard deviation, SED sedentary, LPA light PA, MPA moderate PA, VPA vigorous PA, VVPA very-vigorous PA

The median age of the study sample was 57 years, and the range was 50.1–65.5 years. The sample was stratified into tertiles according to their fitness levels (low, medium, and high). Fitness tertile limits were 34.0 mL/min/kg and 39.3 mL/min/kg for men and 28.1 mL/min/kg and 33.4 mL/min/kg for women. The low fitness group overall had the least favorable cardiometabolic health indicators, and the high fitness group had the most favorable cardiometabolic health indicators (Table 1). In addition, the mean age was 58.4, 57.2, and 56.0 years for the low, medium, and high fitness group, respectively. The means of the standardized PA intensity spectrum variables are presented for each fitness group in Fig. 1. The PA patterns show that the low fitness group was more sedentary and less active at all absolute intensity levels, whereas the intensity pattern of the high fitness group was the opposite. Relative intensity PA generally displayed reversed group differences with the low fitness group being most active; however, in the vigorous intensity range, the high fitness group was still more active than the other groups.

**Table 1:** Characteristics of the whole study sample and by fitness level tertiles. Mean (standard deviation)

			**Fitness group**	
	**Overall**	**Low**	**Medium**	**High**
N (% female)	4176 (51.7%)	1392 (51.9%)	1392 (51.4%)	1392 (51.8%)
Age (years)	57.2 (4.3)	58.4 (4.3)	57.2 (4.2)	56.0 (4.0)
Waist (cm)	92.4 (12.0)	101.0 (10.9)	91.8 (9.5)	84.3 (8.9)
HDL (mmol/L)	1.72 (0.52)	1.55 (0.46)	1.72 (0.51)	1.88 (0.53)
Triglycerides (mmol/L)	1.17 (0.97)	1.43 (1.38)	1.15 (0.71)	0.94 (0.55)
HbA1c (mmol/mol)	35.0 (5.0)	36.1 (6.4)	34.7 (4.2)	34.2 (3.8)
SBP (mmHg)	121.2 (16.3)	126.3 (16.6)	121.4 (15.8)	115.9 (14.8)
Fitness (mL/min/kg)	33.9 (6.7)	27.5 (4.0)	33.6 (3.3)	40.6 (4.6)
Valid days	7.2 (1.3)	7.1 (1.2)	7.3 (1.3)^i^	7.2 (1.2)^i^
Non-wear (minutes/day)	561.4 (93.0)	572.5 (95.8)	558.2 (94.0)^i^	553.6 (88.1)^i^
Absolute (minutes/day)				
Sedentary	680.4 (96.5)	685.4 (100.3)^i^	681.6 (96.6)^i^	674.1 (92.2)
Light PA	126.8 (38.6)	119.3 (38.0)	127.7 (38.9)	133.2 (37.7)
Moderate PA	69.8 (25.1)	62.3 (23.1)	71.3 (25.0)	75.7 (25.1)
Vigorous PA	1.5 (3.7)	0.5 (1.6)	1.1 (2.5)	2.9 (5.3)
Very-vigorous PA	0.2 (1.3)	0.0 (0.3)	0.1 (0.8)	0.5 (2.0)
Relative (minutes/day)	*individual fitness*	*group mean fitness*		
Sedentary^a^	680.4 (96.5)	685.4 (100.3)^i^	681.6 (96.6)^i^	674.1 (92.2)
Light PA	185.4 (55.6)	162.9 (49.2)	192.3 (52.8)	207.9 (53.1)
Moderate PA	11.0 (13.9)	17.9 (13.2)	6.9 (8.3)	2.6 (4.1)
Vigorous PA	1.7 (4.1)	1.1 (2.2)	0.8 (2.1)	1.8 (4.4)
Very-vigorous PA	0.1 (0.7)	0.2 (1.2)^i^	0.2 (1.0)^i^	0.1 (0.4)

^a^Since no established relative cut off for being sedentary is available, the absolute cut-off at 1.5 METs was used for relative sedentary time. ^i^Nonsignificant difference between fitness groups (post hoc t-test). All other group comparisons are significant at *p* < 0.05

*HDL* High-density lipoprotein, *HbA1c* Glycated hemoglobin, *SBP* Systolic blood pressure, *PA* Physical activity

### Association between physical activity intensity and cardiometabolic health

**Fig. 2:**
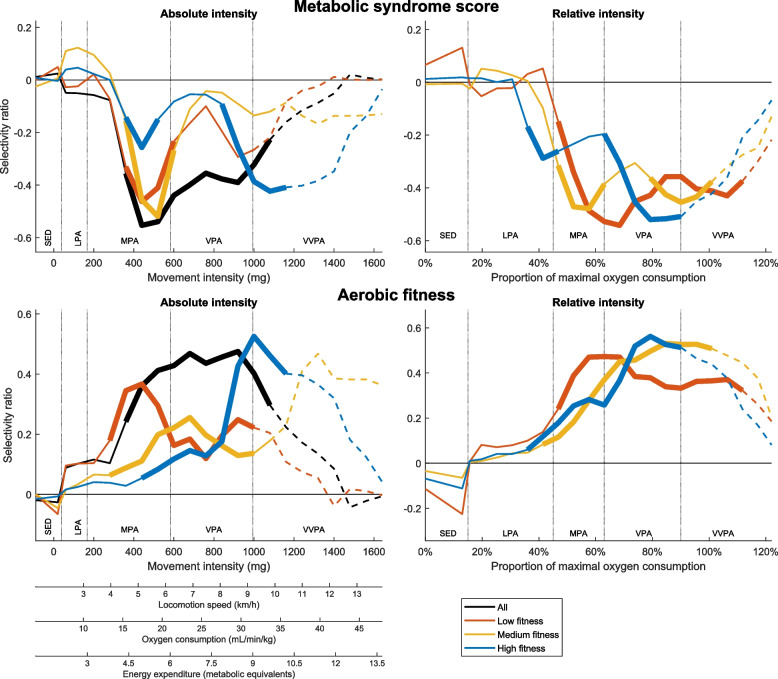
The absolute (left) and relative (right) PA intensity patterns associated with metabolic syndrome score (top) and fitness (bottom). The selectivity ratio represents the influence of each PA intensity level in the association with the outcome. The thick lines represent the main statistically significant part based on 95% confidence intervals, and the dashed lines indicate where less than one third of the individuals had any movement. Since no established relative cut off for sedentary is available, the absolute cut off at 1.5 METs was used as reference for relative sedentary time based on the average fitness in the sample. SED sedentary, LPA light PA, MPA moderate PA, VPA vigorous PA, VVPA very-vigorous PA

In terms of absolute PA intensity, the results of the PLS analysis of the whole study sample show that there was a significant association between PA intensity and the metabolic syndrome score and fitness from the mid-moderate PA range to the lower part of the very-vigorous PA range (Fig. 2, left panels). However, the results of the fitness stratified PLS analyses show that significant associations only in the moderate PA intensity range in the low fitness group. In the medium fitness group, the strongest associations were in the moderate PA range for metabolic syndrome score and in the vigorous PA range for fitness. In the high fitness group, the strongest associations were around the vigorous to very-vigorous cut-off for both health indicators.

In terms of relative PA intensity, the results of the PLS analyses show that the intensities related to metabolic syndrome score and fitness were more synchronized between fitness groups (Fig. 2, right panels). The lowest PA intensity that was significantly associated with the metabolic syndrome score and fitness was close to the relative moderate cut-off at 46% of maximal oxygen consumption for all fitness groups. Furthermore, the strongest association was apparent in the vigorous PA range corresponding to between 64 and 91% of maximal oxygen consumption. The association weakened in the very-vigorous PA range, presumably due to excessive zero score.

All PLS models were statistically significant with *p* < 0.01. More details regarding the PLS models are found in Supplementary Table 2 in Additional file 1.

**Fig. 3:**
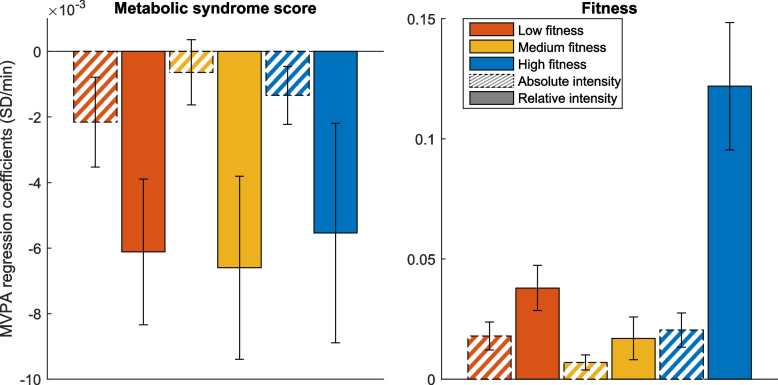
Dose–response relationship from time spent at absolute or relative moderate-to-vigorous physical activity (MVPA) as independent variable and metabolic syndrome (left) and fitness (right) as dependent variable in each of the three fitness groups. Coefficients from regression models are expressed as number of standard deviations difference in the health variable from one minute increase in time spent at MVPA per day. Error bars indicate 95% confidence intervals

With coarse measures of PA, representing time spent at moderate-to-vigorous intensity, the linear regression coefficients from relative moderate-to-vigorous intensity were significantly larger than their absolute intensity counterparts for both fitness and cardiometabolic health indicators (Fig. 3). PA associated more strongly with fitness than with the metabolic syndrome score, especially relative intensity in the high fitness group.

### Fulfilment of physical activity recommendations

In the whole study sample, the mean length of time spent at absolute moderate-to-vigorous intensity was 500 (95% confidence interval 495–506) minutes per week, and 99% (99–100%) fulfilled the recommendations of at least 150 min of moderate-to-vigorous PA intensity per week. When relating PA intensity to individual fitness, the mean moderate-to-vigorous PA level was 91 (87–94) minutes per week and 21% (20–22%) fulfilled the PA recommendations. Furthermore, when relating moderate PA intensity to a sufficiently high fitness of 31.5 and 35 mL/min/kg for women and men, respectively, [29] the mean moderate-to-vigorous PA level was 56 (54–58) minutes per week and only 9% (8–10%) fulfilled the PA recommendations.

**Fig. 4:**
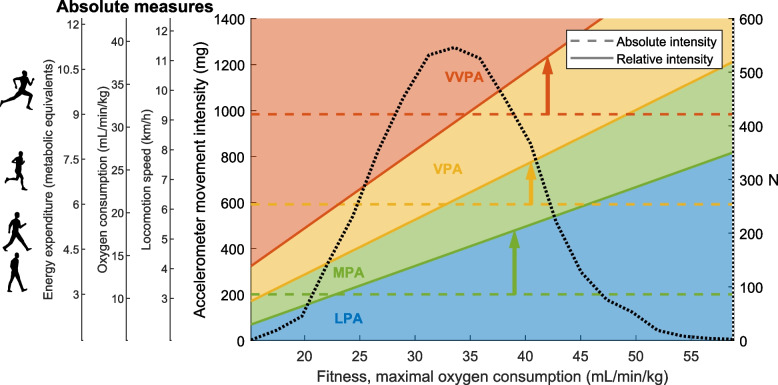
Translation between absolute and relative PA intensity levels. Individual fitness level on the x-axis and different measures of absolute intensity on the y-axis. Background colors with solid borders represent relative intensity. Dashed lines denote absolute intensity. The black dotted line represents the distribution of fitness in the study sample. The intersects between the dashed and solid lines represent the fitness level where the absolute and relative PA intensity is congruent. In individuals with a higher fitness than this, time spent at different intensities will be overestimated, which is emphasized by the colored arrows. For example, a relatively unfit individual with a maximal oxygen consumption of 23 mL/min/kg (x-axis) will have a relative MPA accelerometer cut point at 201 mg (y-axis) which corresponds to a locomotion speed of 3.2 km/h (slow walking) and a MET of 3.0 (at the intersection of the green dashed and solid lines). For comparison, an individual with a higher maximal oxygen consumption of 35 mL/min/kg will have a relative MPA accelerometer cut point at 388 mg which corresponds to a locomotion speed of 4.8 km/h (brisk walking) and a MET of 4.6. If the cut point of the unfit individual (MET of 3.0) is applied to the individual with higher fitness, the time spent in MPA is overestimated. LPA, light PA, MPA moderate PA, VPA vigorous PA, VVPA very-vigorous PA

Figure 4 visualizes the translation between absolute and relative intensity. The intersections between the dashed and solid lines represent the fitness level where absolute intensity corresponds to relative intensity. For moderate intensity, this intersection is at the 5^th^ percentile, suggesting that the absolute intensity cut-off is too low for 95% of the sample. The vigorous and very-vigorous intersections are at the 45^th^ and 55^th^ percentiles, respectively, which are more representative of the average fitness level in the sample.

## Discussion

### Health-related physical activity intensity

In this study, we investigated associations between accelerometer-measured absolute and relative PA intensities and cardiometabolic health in a subgroup of participants from SCAPIS. A key finding was that relative PA intensity, determined from accelerometer data and submaximal fitness test in combination, identified the level that was most strongly associated with cardiometabolic health across all fitness levels, whereas the level of absolute PA intensity associated with cardiometabolic health shifted depending on fitness level. Furthermore, when stratifying for fitness level, the PA intensity patterns of the associations found in this study seem to align with the relative cut-offs of moderate and vigorous intensity that have been suggested based on previous intervention and physiological studies [2, 5]. In relative terms, moderate PA intensity was required to observe significant associations with cardiometabolic health, and associations between PA intensity and cardiometabolic health were stronger for vigorous intensity.

Our results also showed that expressing PA intensity associated with cardiometabolic health in absolute terms was misleading for most individuals in this sample of adults aged 50–64 years. In particular, the absolute moderate intensity cut-off was too low for 95% of the individuals. This implies that relative moderate-to-vigorous PA was considerably more intense than absolute moderate-to-vigorous PA for most individuals and subsequently explains the significantly stronger dose–response relationship with cardiometabolic health and less time spent at this intensity.

In relative terms, the low fitness group was most active overall, which is in line with previous research [13, 14]. However, the detailed analyses in this study showed that the high fitness group was most active at relative vigorous intensity. This might partly explain why this group had a higher fitness level than the low fitness group, despite spending much less time at relative moderate intensity. However, we cannot exclude the possibility that a higher, possibly genetically predisposed, fitness allows for more time spent at higher absolute intensities and that fitness and cardiometabolic risk (e.g. obesity) have shared genetic factors [34].

The differences in associations between absolute and relative PA and cardiometabolic health may explain some of the controversies apparent in previous research regarding the intensity of PA required to provide health benefits. Some previous studies have suggested that accelerometer-measured absolute light intensity PA is associated with cardiometabolic health [8, 9]. These studies typically include older individuals who are less healthy and have lower fitness than in our study sample. On the contrary, studies including healthy young individuals have suggested that absolute moderate intensity PA is not sufficient for associations with health, but that absolute vigorous intensity is required [10]. Thus, for older individuals with low aerobic fitness, absolute light intensity PA could be classified as relative moderate intensity PA, whereas for younger individuals with high aerobic fitness, relative moderate intensity PA may require absolute vigorous intensity PA. Similarly, this could also explain why previous studies in individuals with low fitness suggest an inverse association between sedentary time and health [35]. Time spent sedentary is negatively correlated with time spent at light intensity [36]. This means that an association between sedentary time and health could hypothetically be due to an association between light intensity PA and health, which in turn could be considered relative moderate intensity in individuals with low fitness.

### Recommendations on physical activity

The results of this study suggest that absolute and relative intensity cannot be used interchangeably for most individuals when analyzing accelerometer data, in contrast to the current recommendations on PA [1, 2]. Instead, more emphasis should be put on the relative intensity of a given activity, for example using self-perceived exertion of PA rather than on specific activities at absolute intensities (e.g., “a substantial increase in breathing rate” instead of “brisk walking”).

If an individual is sufficiently physically active to increase fitness, the absolute intensity required to improve fitness further will also increase. Given that the health benefits of increased fitness might level off at a sufficiently high fitness level of 31.5 and 35 mL/min/kg for women and men, respectively, [29] general PA recommendations should be developed with the long-term goal to achieve these fitness levels in a middle-age population. Absolute moderate intensity would then be considered to be above 4.1 and 4.6 METs for men and women, respectively, [29] substantially higher than the current recommendations of 3 METs. The mean fitness level of 33.9 mL/min/kg in the whole sample in our study is close to the suggested sufficient fitness level and implies that these cut-points would also represent health beneficial PA intensity in this sample. These cut-points are also similar to previous research regarding accelerometer-measured moderate intensity [11, 15]. The message in the current recommendations that every move counts is, however, still important. To improve their health and fitness, individuals with low fitness should start with PA intensity that is light on the absolute scale but corresponds to moderate on the relative scale, and then progress to more intense PA. These findings may further support individualized exercise prescription and monitoring, where relative rather than absolute intensity levels are of importance for maintenance and adherence.

### Limitations

The cross-sectional study design does not allow causality to be determined. The results do not directly show which PA intensity level is most beneficial for interventions, but rather which intensity level is associated with health outcomes within each fitness group. Additionally, the results should not be interpreted as showing that individuals with low fitness would not gain health benefits from very high intensity exercise. The weaker association at absolute vigorous intensity is rather explained by very few low fit individuals performing this kind of activity.

The age span of the whole study sample was limited and the participants had a more favorable health status than in other large-scale studies [37, 38]. The traditional absolute moderate intensity cut-off at 3 METs might be more appropriate for another population with less favorable health and lower fitness level. In that case, the absolute vigorous cut-off would instead be too high according to the results of this study. In addition, the studied sample displayed slightly more favorable cardiometabolic health indicators compared to the entire SCAPIS study. This is presumably due to the exclusion criteria of the fitness test, which likely exclude individuals with low fitness to a larger degree. However, these differences in cardiometabolic health indicators are generally smaller than the differences between fitness tertiles. The entire SCAPIS study has been shown to have only minimal selection bias in relation to the Swedish population [39].

The current methods used for processing raw accelerometer data have been shown to capture PA intensity more accurately, [3, 18, 19] but typically result in more time spent at moderate-to-vigorous intensity compared to traditional processing and cut-points [19, 40]. This explains the very high fulfilment of absolute PA recommendations when using absolute intensity with crude moderate-to-vigorous cut-offs by accelerometry in this study and implies that these levels cannot be directly compared to other studies. However, this emphasizes the benefit of using a more detailed intensity spectrum instead of crude cut-points. Furthermore, this provides additional evidence that absolute moderate intensity is too low to represent health-beneficial PA in relation to the guidelines.

The stratification approach naturally misses variation available in the dataset, which weakens the overall association in the PLS models. Because of the normal distribution of fitness in the sample, there is substantially less variation in fitness in the medium fitness group. Furthermore, this study solely focuses on the effect of different PA intensities and assumes that the recommendations of 150 min per week of moderate-to-vigorous intensity PA is applicable to objectively measured PA. Future studies should incorporate the results of this study regarding PA intensity and investigate the effects of different PA volume.

### Implications

Although the results of this study suggest that health-beneficial PA intensity is relative to individual fitness, the results from measurement of relative PA intensity should be interpreted with caution. In cross-sectional studies, a consideration of individual relative intensity PA is comparable to including fitness as a covariate and controlling the association between PA and health outcomes for fitness level. This distorts potential associations because it usually suggest low fit individuals are the most active [12–14]. In intervention studies, however, the effect of PA is more likely to be better represented by relative intensity than by absolute intensity [2, 5]. In these settings, accelerometer-measured individual relative intensity can be directly applied to study the effect of an intervention on PA level or potential effects of PA level on other outcomes.

Instead of considering individual relative intensity in cross-sectional studies, PA intensity relative to the sample mean could be considered. This implies that the cut-points used for analyzing accelerometer data are adjusted to represent health-beneficial PA intensity in the sample studied. This can be done based on the relative cut-points of 46%, 64% and 91% of fitness level and is visualized in Fig. 4. In the present study sample, with an average fitness level of 33.9 ml/min/kg, the cut-points used would be 4.5, 6.2 and 8.8 METs for moderate, vigorous, and very-vigorous PA intensity respectively (e.g., 0.46 × 33.9 = 15.6 ml/min/kg; 15.6 / 3.5 = 4.5 METs). This is equivalent to 391, 618 and 959 mg based on the regression coefficients presented in the methods section (e.g., (0.46 × 33.9 – 5.108) / 0.02683 = 391 mg). As another example, in patients with heart failure and a fitness level of 14.1 ml/min/kg, [41] the corresponding cut-points would be 1.9, 2.6 and 3.7 METs for moderate, vigorous and very-vigorous PA. In this example, even absolute light intensity could be considered relatively vigorous. Clearly, there is a large discrepancy between these cut-points, which emphasizes the need for adjusting them to be relevant for the sample studied. The same adjustments can be used on ActiGraph counts by applying the regression equations for the widely used cut-points from e.g. Freedson et al. [42]. However, the use of relative intensity cut-points limits the comparability of PA levels between studies.

For precise interpretation and prescription of PA intensity, maximal or submaximal fitness tests would ideally be considered in both research and clinical practice. Alternatively, when fitness tests are not available, fitness level may be estimated from age, sex and self-perceived physical capacity [43]. To aid in clinical practice, we provide a tool for translation between relative and absolute intensity (Fig. 4), to be considered when interpreting research on physical activity or when prescribing PA as treatment.

## Conclusions

Our study suggests that accelerometer-measured relative moderate-to-vigorous PA intensity represents the PA intensity associated with health regardless of fitness level. Absolute moderate PA intensity represents health-associated PA only in a small proportion of individuals. Relative intensity cut-offs representing moderate and vigorous PA intensity align with the associations to health indicators observed in this study when applied to accelerometer data. Traditional absolute moderate intensity PA accelerometer cut-offs are too low for most individuals and should be adopted to individual fitness or the fitness level in the sample studied. Absolute and relative PA intensity cannot be used interchangeably in PA recommendations, and more emphasis should be put on relative intensity, for example by using self-perceived exertion, when communicating the PA intensity required to benefit health.

### Supplementary Information

**Additional file 1: Table 1. **Characteristics of the study sample, individuals from the Gothenburg site excluded due to missing measurement of fitness and PA, and the entire SCAPIS sample. Mean (standard deviation). **Table 2.** PLS model details. Number of PLS components was chosen based on cross validation.

## Data Availability

The data that support the findings of this study are available from the SCAPIS study organization (www.scapis.org) but restrictions apply to the availability of these data, which were used under license for the current study, and so are not publicly available. Data are however available from the authors upon reasonable request and with permission of the SCAPIS study organization.

## Abbreviations

HbA1c: Glycated hemoglobin
HDL: High density lipoprotein
METs: Metabolic equivalents of task
PA: Physical activity
PLS: Partial least squares regression
SBP: Systolic blood pressure
SCAPIS: Swedish cardiopulmonary bioimage study
