# Supplementary material for: Accelerometer-measured absolute versus relative physical activity intensity: cross-sectional associations with cardiometabolic health in midlife
Source: BMC Public Health. 2023 Nov 24;23:2322. doi: 10.1186/s12889-023-17281-4 (PMC10668340; doi:10.1186/s12889-023-17281-4)
Supplement: Supplementary file 1 — Additional file 1: Table 1. Characteristics of the study sample, individuals from the Gothenburg site excluded due to missing measurement of fitness and PA, and the entire SCAPIS sample. Mean (standard deviation). Table 2. PLS model details. Number of PLS components was chosen based on cross validation. [file 12889_2023_17281_MOESM1_ESM.docx]

**Supplementary Table 1.** Characteristics of the study sample, individuals from the Gothenburg site excluded due to missing measurement of fitness and PA, and the entire SCAPIS sample. Mean (standard deviation).

|  | **Study sample** | **Excluded (missing data)** | **Entire SCAPIS** |
| --- | --- | --- | --- |
| N (% female) | 4176 (51.7%) | 1945 (52.5%) | 29775 (51.3%) |
| Age (years) | 57.2 (4.3) | 58.1 (4.3) | 57.5 (4.3) |
| Waist (cm) | 92.4 (12.0) | 96.0 (14.2) | 94.4 (12.9) |
| HDL (mmol/L) | 1.72 (0.52) | 1.60 (0.50) | 1.63 (0.50) |
| Triglycerides (mmol/L) | 1.17 (0.97) | 1.38 (1.16) | 1.24 (0.83) |
| HbA1c (mmol/mol) | 35.0 (5.0) | 36.8 (7.3) | 36.6 (6.5) |
| SBP (mmHg) | 121.2 (16.3) | 125.19 (17.4) | 125.90 (17.0) |

Only individuals with valid measurements of all metabolic health indicators are presented. All comparisons between the study sample and the other groups were significant at p < 0.05 (independent samples t-test). HDL, high-density lipoprotein; HbA1c, glycated hemoglobin; SBP, systolic blood pressure; PA, physical activity.

**Supplementary Table 2.** PLS model details. Number of PLS components was chosen based on cross validation.

| **Outcome** | **Group** | **Intensity** | **Number of PLS components** | **Explained variance in the outcome** |
| --- | --- | --- | --- | --- |
| Fitness | Overall | Absolute | 2 | 24.6% |
|  | Low fitness | Absolute | 1 | 5.2% |
|  |  | Relative | 1 | 4.4% |
|  | Moderate fitness | Absolute | 1 | 2.4% |
|  |  | Relative | 1 | 2.7% |
|  | High fitness | Absolute | 1 | 7.9% |
|  |  | Relative | 1 | 10.0% |
| Metabolic syndrome score | Overall | Absolute | 2 | 12.4% |
|  | Low fitness | Absolute | 1 | 3.0% |
|  |  | Relative | 1 | 3.3% |
|  | Moderate fitness | Absolute | 1 | 1.7% |
|  |  | Relative | 1 | 2.0% |
|  | High fitness | Absolute | 1 | 2.8% |
|  |  | Relative | 1 | 3.0% |
